# Supplementary material for: Dynamic Structural Recovery Parameters Enhance Prediction of Visual Outcomes After Macular Hole Surgery
Source: Transl Vis Sci Technol. 2025 Dec 29;14(12):29. doi: 10.1167/tvst.14.12.29 (PMC12758438; doi:10.1167/tvst.14.12.29)

**Supplementary Data**

**Supplementary Figure 1**. Precision-Recall Curve (AUPRC) comparing the predictive performance of deep learning (DL) and logistic regression (LG) models across different data modality combinations, with or without dynamic parameters (DP), for postoperative best-corrected visual acuity (BCVA) improvement at four timepoints, (A) 2 weeks, (B) 3 months, (C) 6 months, and (D) 12 months after surgery. CD means clinical data; Values means extracted structural features. Across all stages, the DL model integrating CD, Values (including DP), and images achieved the highest AUC. Exclusion of DP led to a consistent decline in performance, particularly in mid-term prediction. This result is basically consistent with the ROC-AUC prediction result.


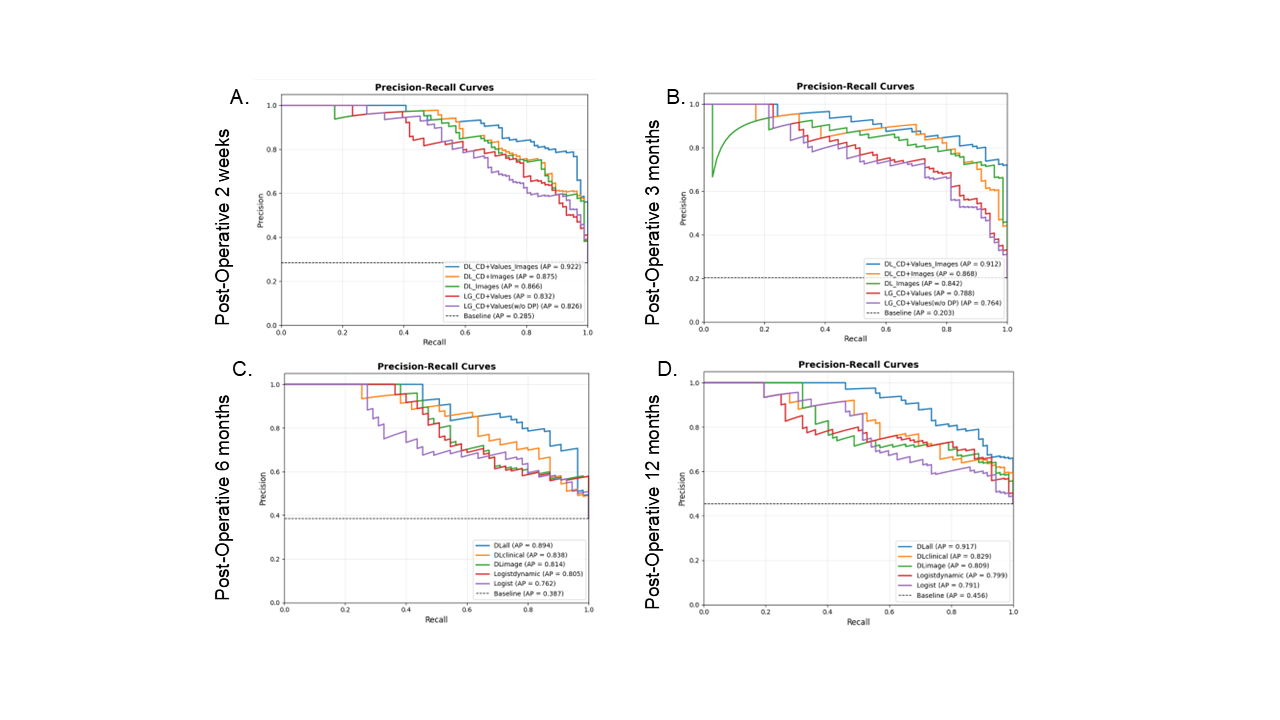

Supplement: Supplement 1 [file tvst-14-12-29_s001.docx]
